# Timing Tasks Synchronize Cerebellar and Frontal Ramping Activity and Theta Oscillations: Implications for Cerebellar Stimulation in Diseases of Impaired Cognition

**DOI:** 10.3389/fpsyt.2015.00190

**Published:** 2016-01-18

**Authors:** Krystal L. Parker

**Affiliations:** ^1^Department of Neurology, Carver College of Medicine, University of Iowa, Iowa City, IA, USA

**Keywords:** eyeblink conditioning, interval timing, ramping activity, theta oscillations, cerebellum, prefrontal cortex

## Abstract

Timing is a fundamental and highly conserved mammalian capability, yet the underlying neural mechanisms are widely debated. Ramping activity of single neurons that gradually increase or decrease activity to encode the passage of time has been speculated to predict a behaviorally relevant temporal event. Cue-evoked low-frequency activity has also been implicated in temporal processing. Ramping activity and low-frequency oscillations occur throughout the brain and could indicate a network-based approach to timing. Temporal processing requires cognitive mechanisms of working memory, attention, and reasoning, which are dysfunctional in neuropsychiatric disease. Therefore, timing tasks could be used to probe cognition in animals with disease phenotypes. The medial frontal cortex and cerebellum are involved in cognition. Cerebellar stimulation has been shown to influence medial frontal activity and improve cognition in schizophrenia. However, the mechanism underlying the efficacy of cerebellar stimulation is unknown. Here, we discuss how timing tasks can be used to probe cerebellar interactions with the frontal cortex and the therapeutic potential of cerebellar stimulation. The goal of this theory and hypothesis manuscript is threefold. First, we will summarize evidence indicating that in addition to motor learning, timing tasks involve cognitive processes that are present within both the cerebellum and medial frontal cortex. Second, we propose methodologies to investigate the connections between these areas in patients with Parkinson’s disease, autism, and schizophrenia. Lastly, we hypothesize that cerebellar transcranial stimulation may rescue medial frontal ramping activity, theta oscillations, and timing abnormalities, thereby restoring executive function in diseases of impaired cognition. This hypothesis could inspire the use of timing tasks as biomarkers for neuronal and cognitive abnormalities in neuropsychiatric disease and promote the therapeutic potential of the cerebellum in diseases of impaired cognition.

## Introduction

Timing is highly conserved for all mammals, and although it is paramount to survival, the precise neural mechanisms underlying the perception of time are unknown. Depending on the duration of time and type of behavioral task, the frontal cortex, striatum, hippocampus, and the cerebellum have been implicated in timing (1, 2). Neuropsychiatric illnesses such as Parkinson’s disease (PD), autism, and schizophrenia involve cognitive impairment (3, 4). Mammals depend on time for working memory, attention, reasoning, communication, decision-making, and movement. As a valid proxy for cognition, timing tasks present a window into aberrant neural circuitry in animal models and in human neuropsychiatric disease (4–6).

The seminal theories of cognitive dysfunction in neuropsychiatric disease indicate a disruption in the fluid and coordinated sequences of thought and action that are the hallmarks of normal cognition (7, 8). Based on consistent abnormalities in structural and functional imaging of schizophrenia, cognitive dysmetrias are thought to occur as a result of abnormalities in a network between the cerebellum and frontal cortex (7, 8). The network connecting the frontal cortex and cerebellum involves an efferent disynaptic projection via corticospinal tracts to the ipsilateral rostral pontine nuclei (9). The afferent cerebellar projection is through the ventrolateral and mediodorsal thalamic nuclei (10–13). Cerebellar stimulation dynamically influences the medial frontal cortex in animals (14–16) and is safe and effective in alleviating cognitive impairments and elevating mood in patients with schizophrenia (17). Therefore, the pathway between the cerebellum and medial frontal cortex could be isolated to investigate cognitive circuitry and the therapeutic potential for cerebellar stimulation in diseases involving compromised cognition.

## Timing Tasks Require Cognitive Processing in the Cerebellum and Frontal Cortex

Eyeblink conditioning and interval timing are two tasks requiring temporal processing that can be used in animals and humans to investigate the cerebellar influence on the frontal cortex. Eyeblink conditioning is the canonical paradigm to investigate cerebellar function as timing is impaired following cerebellar inactivation and lesion (18–24). Additionally, eyeblink conditioning is a powerful technique to illuminate cerebellar dysfunction in neuropsychiatric disorders (25–28). Eyeblink conditioning involves the pairing of a neutral conditioned stimulus (CS), such as a light or tone, with an aversive unconditioned stimulus (US), typically an airpuff to the eye or periorbital shock, to elicit an unconditioned response (UR). Following repeated pairings of the CS and US, the subject adaptively predicts the pending US and elicits a preventative conditioned eyeblink response (CR) that precedes the onset of the US. Two types of eyeblink conditioning exist in which there is either no interval between the CS and US and the two stimuli co-terminate (delay conditioning) or an interval of time between the two so that the offset of the CS is several milliseconds or seconds before the onset of the US (trace conditioning). Although studies claim trace and delay conditioning recruit different brain regions, they both involve activity in the cerebellum and medial frontal cortex (29–31).

This is an important consideration because the cerebellum and medial frontal cortex are both essential for accurate timing and both are aberrant in neuropsychiatric disease (25–27, 30–34). Although eyeblink conditioning involves motor performance, timing the interval also requires working memory, attention to time, and therefore involves cognitive processing. Animals with a disrupted cerebellum (35) and humans with cerebellar damage exhibit spared motor performance while eyeblink conditioning is impaired (36), indicating a separate role of the cerebellum in cognitive and motor function. Additionally, PET imaging studies indicate that both the frontal cortex and cerebellum are involved in eyeblink conditioning (37, 38) and they are hypoactive concurrent with impairments in eyeblink conditioning in patients with schizophrenia (26, 27).

Interval timing closely resembles eyeblink conditioning in that two stimuli are separated by an interval of time, and subjects estimate the passage of the specified interval. Subjects hold temporal information regarding the passage of time in their mind while they estimate when the respective amount of time has elapsed by making a motor response. Interval timing critically depends on the medial frontal cortex, which is impaired in patients with neuropsychologic illness (25–27, 30–34). There are currently no studies in animals reporting a cerebellar involvement in interval timing, likely due to the traditional view of cerebellar contributions to only subsecond temporal processing (2). However, humans with cerebellar damage have profound deficits discriminating longer intervals (8–32 s) in a temporal bisection task (39). Therefore, the cerebellum merits further investigation during interval timing tasks that require timing in the range of seconds. By combining interval timing literature with the work on eyeblink conditioning, we could gain insight into the function of cingulocerebellar circuitry and its dysfunction in cognitive disease.

## Timing Tasks Can be Used to Probe the Neural Mechanisms Underlying Cognitive Processing

Although different timescales are often used, there are two types of neuronal activity that are consistently described during timing tasks: ramping (consistent increases or decreases in neuronal firing) (40–48) and low-frequency oscillations (42, 43, 49, 50). Single medial frontal cortical neurons that are consistently active or increase or decrease activity to bridge the interval between the CS and US are consistently reported during operant and classical conditioning paradigms, including eyeblink conditioning (9), interval timing (42), and fear conditioning (51). These neurons are often referred to as climbing, bridging, or ramping neurons, but we will refer to them as ramping neurons in this manuscript.

Ramping activity involves the accumulation of temporal information between the stimuli encoding the start of the trial, US or reward availability, and response time. Of these ramping neurons, 15–20% of them encode the passage of time by ramping or accumulating the increase or decrease in action potentials over a behaviorally relevant timing window (9, 52). Although essential to bridge the CS and US, this activity may indicate when to respond prior to the end of the CS in delay conditioning. A subset of cerebellar neurons shows a similar pattern of bridging or ramping activity to that of frontal neurons during eyeblink conditioning (9, 53, 54). Therefore, it is speculated that consistent activity in the medial frontal cortex provides the cerebellum with timing information for bridging the temporal gap between the CS and US regardless of the presence of an interstimulus interval (9).

Ramping activity that reverberates throughout the circuit could represent timing as a circuit-wide phenomenon rather than structure and task specific. Investigating concurrent medial frontal and cerebellar activity during timing tasks in healthy and aberrant states could elucidate how the brain encodes cognitive processes. Neuronal activity that lapses the interval could represent working memory processes. Therefore, combining the literature from both the eyeblink conditioning and interval timing fields could provide a circuit-based interpretation of how the brain encodes time and incidentally, cognition.

In addition to the role of ramping activity during timing, cue-evoked theta activity is also essential for temporal processing (42). During interval timing, rodents and humans have similar bursts of low-frequency activity immediately following trial start (42, 43) as measured by multi-neuron local field potential (LFP) signals. This burst of cue-evoked activity could represent the start of an internal clock in timing tasks that initiates ramping activity in single neurons to encode the passage of time (50). Low-frequency oscillations also synchronize activity within brain networks as revealed by coherence in theta frequencies between brain areas, presenting a mechanism for how neuronal networks organize behavior across time (55).

Concomitant with ramping patterns, medial frontal theta activity is dependent on dopamine as revealed by diminution of low-frequency oscillations following focal D1 dopamine blockade in the frontal cortex during interval timing (42, 43). PD characteristically involves dopamine dysfunction, and consistent with these results, medial frontal theta activity is attenuated in PD patients (43). We previously described common mid-frontal oscillations triggered by the cue (tone) during interval timing tasks in both humans and rodents (43). Additionally, the prelimbic cortex and cerebellar nuclei are coupled at low frequencies (56, 57). Synchronization between ramping neurons in both the cerebellum and frontal cortex during cognitive processing indicates that rather than one area encoding time, low-frequency activity throughout a circuit may be essential, implicating a highly conserved neural architecture for temporal organization of behavior in mammals.

## Cerebellar Stimulation during Timing Tasks Can be Used to Rescue Neural Mechanisms Underlying Cognition

If cerebellar and frontal areas both encode cognitive processes, cerebellar stimulation could be used to recover aberrant neuronal activity and rescue cognitive abnormalities in disease. Cerebellar vermal transcranial magnetic stimulation (TMS) produced downstream changes in neuronal activity in the frontal cortex as revealed by electroencephalogram (EEG) (58). A classic study by Cooper et al. electrically stimulated the cerebellum in patients with epilepsy and reported improved cognition based on increased alertness, improvement in thinking, and fluency of speech in addition to many enriched emotional characteristics (59). Recently, cerebellar theta-burst (TMS) was reported to be safe and effective in alleviating some cognitive impairments and elevating mood in treatment-resistant schizophrenia patients (17). There are currently several clinical trials further investigating the therapeutic potential of the cerebellum in schizophrenia, yet the underlying neuronal mechanisms remain unknown – Clinicaltrials.gov (60). These studies indicate that there is great potential for cerebellar stimulation to be used to treat cognitive symptoms of neuropsychiatric disease pending the explicit mapping and understanding of the influence of the cerebellum on frontal circuits.

Cerebellar dentate electrical stimulation has been shown to influence the dopamine efflux in the frontal cortex (14–16, 61). Conversely, electrical stimulation of the prelimbic frontal cortex elicited neuronal firing in cerebellar lobule VII (61) establishing a physiologic mechanism for communication between the two areas. However, to our knowledge, cerebellar stimulation has never been explored in behaving animals. We recently described a novel method to use cerebellar optogenetic stimulation to rescue cognitive deficits induced by pharmacological frontal inactivation in behaving animals. In addition to providing critical information regarding aberrant neural circuitry in disease, cerebellar stimulation can be used to recover dysfunctional neurons and rescue timing impairments in eyeblink conditioning and interval timing tasks.

## Clinical Implications

We have hypothesized that cognitive processing during timing tasks relies on low-frequency, cue-evoked activity in the medial frontal cortex to signal the start of single neuron ramping. Ramping activity could represent an internal clock encoding the passage of time and indicating when to make a motor response (41, 42). By combing frontal EEG with cerebellar TMS, we can investigate how cerebellar stimulation influences neuronal activity in the frontal cortex. We hypothesize that low-frequency cerebellar stimulation will reinstate both low-frequency oscillations and ramping properties of medial frontal neurons in patients with neuropsychiatric illness.

Electroencephalogram activity indicates the sum of a large population of neurons over a relatively poor spatially represented area. This technique will allow us to investigate neuronal oscillations in humans, but only rodent models can be used to investigate how stimulation influences ramping activity. We recently explored temporal processing in PD (43). PD involves the death of dopaminergic neurons in the substantia nigra, pars compacta and in the ventral tegmental area that projects to the frontal cortex (62). We hypothesized that dysfunctional frontal dopamine would lead to diminished frontal theta and result in impaired interval timing performance. We recorded EEG from patients with PD and healthy controls while they performed interval timing tasks, and to explore ramping activity, we used an animal model of frontal dopamine depletion with 6-OHDA in the medial frontal cortex. Interestingly, patients with PD and animal models of PD have diminished oscillations during interval timing tasks and ramping activity is diminished concurrent with dysfunctional temporal processing (43). These data indicate specific dopamine-dependent activity in the medial frontal cortex is necessary for interval timing and therefore, cognitive processing.

We hypothesize that cognitive abnormalities are similar between many neuropsychiatric diseases including PD, schizophrenia, and autism. In schizophrenia, the prefrontal cortex shows abnormal D1 dopamine (63–65), and patients inaccurately estimate time (66, 67). Cerebellar TMS has been shown to decrease negative symptoms including cognitive processing in patients with schizophrenia (17). However, if cerebellar stimulation is to become a useful treatment strategy targeted at currently untreatable cognitive impairments in schizophrenia, the precise neuronal effects of cerebellar stimulation need to be illuminated. Reinhart et al. recently reported that patients with schizophrenia have impaired frontal theta activity and cerebellar stimulation appears to rescue this activity (55, 68). The therapeutic potential of cerebellar stimulation during timing tasks has never been studied. Thus, combined TMS and EEG neural recordings in patients with PD, schizophrenia, autism can be used to investigate the neural mechanisms underlying cognitive processing during timing tasks. Cerebellar stimulation is currently in clinical trials to be used to treat the recurrent cognitive symptoms of schizophrenia. Therefore, we expect that insights from this research will guide future therapies for devastating neuropsychiatric diseases. Performance on timing tasks and frontal dysfunction may be a useful clinical biomarker of frontal dysfunction in neuropsychiatric illness.

## Conclusion

Eyeblink conditioning and interval timing are powerful techniques that can be used in both human and animals to probe cognitive processing in cerebellar and frontal cortical circuitry. Timing tasks can provide us with a behavioral outcome to evaluate the efficacy of cerebellar stimulation on the frontal cortex neuronal activity and cognitive processing neuropsychiatric diseases including schizophrenia, bipolar disorder, ADHD, autism, OCD, and PD. As EEG is widely available, inexpensive, and easily executed, the detection of diminished frontal theta has the potential to be used as a biomarker of neuropsychiatric cognitive and neuronal dysfunction (28). TMS is rapidly becoming an important research tool in neuropsychiatric illness (60), so identifying a specific type of activity that encodes timing and cognition could guide individualized stimulation according to abnormalities in real time. Specifically, a closed-loop design where cerebellar stimulation is based on real time, aberrant frontal activity as defined by a temporal prediction error, could inspire a new paradigm to adaptively stimulate cerebellar neurons using TMS with temporal specificity to reinstate accurate timing and cognitive processes (69).

## Author Contributions

KP takes full authorship of this manuscript.

## Conflict of Interest Statement

The author declares that the research was conducted in the absence of any commercial or financial relationships that could be construed as a potential conflict of interest.
